# Epidemiological Assessment of Oral and Oropharyngeal Squamous Cell Carcinomas Before, During, and After the COVID-19 Pandemic, 2018–2024

**DOI:** 10.3390/jcm15186963

**Published:** 2026-09-08

**Authors:** George Cătălin Alexandru, Călin Muntean, Doina Chioran, Mircea Riviș, Loredana-Neli Gligor, Marius Octavian Pricop, Tudor Rareş Olariu

**Affiliations:** 1 Doctoral School, Victor Babeș University of Medicine and Pharmacy, Eftimie Murgu Square, No. 2, 300041 Timișoara, Romania; george.alexandru@umft.ro (G.C.A.); loredana.alexandru@umft.ro (L.-N.G.); 2 Center for Diagnosis and Study of Parasitic Diseases, Department of Infectious Disease, Victor Babeș University of Medicine and Pharmacy, 300041 Timișoara, Romania; rolariu@umft.ro; 3 Department of Medical Informatics and Biostatistics, Victor Babeș University of Medicine and Pharmacy, Eftimie Murgu Square, No. 2, 300041 Timișoara, Romania; calin.muntean@umft.ro; 4 Department of Anesthesiology and Oral Surgery, Victor Babeș University of Medicine and Pharmacy, Eftimie Murgu Square, No. 2, 300041 Timișoara, Romania; rivis.mircea@umft.ro; 5 Research Center of Dento-Alveolar Surgery, Anesthesia and Sedation in Dental Medicine, University Clinic of Anesthesiology and Oral Surgery, Victor Babeș University of Medicine and Pharmacy, 300041 Timișoara, Romania; 6 Department of Pulmonology, Center for Research and Innovation in Precision Medicine of Respiratory Diseases, Victor Babeș University of Medicine and Pharmacy, Eftimie Murgu Square, No. 2, 300041 Timișoara, Romania; 7 Department of Oral and Maxillofacial Surgery, Victor Babeș University of Medicine and Pharmacy, 300041 Timișoara, Romania; 8 Discipline of Parasitology, Department of Infectious Diseases, Victor Babeș University of Medicine and Pharmacy, 300041 Timișoara, Romania; 9 Clinical Laboratory, Municipal Clinical Emergency Hospital, 300254 Timișoara, Romania; 10 Patogen Preventia, 300124 Timișoara, Romania

**Keywords:** oral squamous cell carcinoma, oropharyngeal cancer, COVID-19 pandemic, head and neck oncology, interrupted time-series, mixed-methods, surgical complexity, maxillofacial surgery

## Abstract

**Background/Objectives:** Evidence regarding changes in oral and oropharyngeal squamous cell carcinoma (SCC) care across the COVID-19 pandemic remains limited, particularly in Eastern Europe. This study evaluated temporal changes in surgical admission volume, operative complexity, and comorbidity burden before, during, and after the pandemic at a Romanian tertiary maxillofacial center. **Methods:** This retrospective mixed-methods study included 248 admissions for oral and oropharyngeal SCC between 2018 and 2024. A structured administrative database was deterministically linked to narrative discharge summaries. Admissions were grouped into pre-pandemic (*n* = 117), pandemic (*n* = 93), and post-pandemic (*n* = 38) periods. Statistical analyses included between-period comparisons, segmented interrupted time-series analysis, and multivariable logistic regression. Qualitative thematic analysis was used to contextualize surgical and perioperative complexity. **Results:** Mean monthly admissions decreased from 4.9 before the pandemic to 2.6 during the pandemic and 1.6 in the post-pandemic period. Interrupted time-series analysis identified a significant declining baseline monthly trend (IRR 0.973, 95% CI 0.950–0.996; *p* = 0.024), without a significant immediate level change or post-onset slope change. Neck dissection increased from 23.9% before the pandemic to 39.8% during the pandemic (*p* = 0.045) and was independently associated with the pandemic/post-pandemic period (adjusted OR 1.87, 95% CI 1.06–3.29; *p* = 0.030). Qualitative findings identified recurrent codes related to surgical complexity, reconstruction, extensive nodal surgery, comorbidity burden, and perioperative management. **Conclusions:** Oral and oropharyngeal SCC surgical admissions showed a significant declining baseline trend between 2018 and 2024, while patients treated during and after the pandemic had higher adjusted odds of undergoing neck dissection. The findings support sustained oncological surveillance, timely referral, and multidisciplinary perioperative planning. Further multicenter studies incorporating standardized tumor–node–metastasis (TNM) staging and human papillomavirus (HPV) documentation are required to confirm these observations.

## 1. Introduction

Head and neck squamous cell carcinoma (HNSCC) represents a major global cancer burden, with the oral cavity and oropharynx accounting for a substantial proportion of cases [1]. Oral and oropharyngeal SCC are epidemiologically heterogeneous: oral-cavity tumors are predominantly associated with tobacco and alcohol exposure, whereas an increasing proportion of oropharyngeal tumors are HPV-related [2,3,4,5]. Timely diagnosis and treatment are particularly important because treatment delays have been associated with more advanced presentation and poorer outcomes [6,7,8,9].

The COVID-19 pandemic disrupted cancer diagnosis and treatment worldwide, including head and neck oncological care [10,11,12,13,14,15,16,17,18,19]. Previous studies reported reductions in surgical activity, delayed referral or treatment, more advanced presentation, and changes in reconstructive requirements during the pandemic [11,12,13,14,15,16,17,18,19]. However, most available studies focused on the early pandemic period or relied primarily on aggregate data, while detailed longitudinal evidence spanning the pre-pandemic, pandemic, and post-pandemic periods remains limited, particularly in Central and Eastern European tertiary centers.

At the regional level, the rapid spread of SARS-CoV-2 in Western Romania was documented by several seroepidemiological studies conducted with the involvement of the Municipal Clinical Emergency Hospital of Timișoara. An initial investigation involving 2115 blood donors reported a seroprevalence of 1.51% in 2020 [20]. Subsequent studies showed a marked increase in seroprevalence, reaching 41.04% among blood donors after the third pandemic wave [21] and 45.60% among individuals undergoing routine laboratory investigations between March and June 2021 [22]. By early 2023, SARS-CoV-2 seroprevalence had reached 88.37% among adult outpatients from Western Romania [23], while a further study conducted among blood donors in August–September 2023 reported a nucleocapsid-antibody seroprevalence of 89.69% [24]. Taken together, these findings demonstrate the rapid and widespread transmission of SARS-CoV-2 in the population served by the study center and provide an important regional context for assessing its impact on oncological services.

We therefore aimed to: (1) quantify the volume, demographic profile and temporal dynamics of oral and oropharyngeal SCC admissions across the pre-pandemic, pandemic and post-pandemic periods, including interrupted time-series modeling; (2) characterize surgical complexity (extent of resection, neck dissection, reconstruction), histopathological proxies of advanced disease and comorbidity burden, and test for changes across periods; and (3) identify independent predictors of neck dissection as a marker of complex nodal surgery.

## 2. Materials and Methods

### 2.1. Study Design and Setting

We conducted a single-center, retrospective, mixed-methods study at the Clinic of Oral and Maxillofacial Surgery (OMFS), Timișoara, a tertiary referral center that serves the historical Banat region and adjacent counties of western and south-western Romania (catchment population ≈ 2.5 million). The study followed an explanatory sequential logic in which a quantitative epidemiological arm was complemented and contextualized by a qualitative thematic arm derived from the same encounters. Reporting adheres to the STROBE statement for observational studies and to good practice for mixed-methods health research. No questionnaires or survey instruments were used in this study. The quantitative and qualitative components were derived from the same linked clinical encounters and were used for complementary rather than interchangeable purposes: structured administrative data were used to quantify temporal and epidemiological patterns, whereas narrative discharge summaries provided detailed clinical, operative, and pathological information and supported contextual qualitative interpretation.

### 2.2. Ethics Statement

The study was conducted in accordance with the Declaration of Helsinki and the principles of good practice in biomedical research. Ethical approval was granted by the Scientific Research Ethics Committee of the “Victor Babeș” University of Medicine and Pharmacy, Timișoara (Approval No. 94/04.10.2021, revised in 2025). The approved project included the assessment of diagnostic and treatment delays, advanced-stage presentation, and increased surgical complexity in patients with oral and maxillofacial malignancies during the COVID-19 pandemic. The requirement for individual informed consent was waived owing to the retrospective design of the study and the exclusive use of fully de-identified administrative and clinical records, in accordance with institutional policy and applicable data-protection regulations, including the General Data Protection Regulation (GDPR).

### 2.3. Data Sources and Record Linkage

**Figure 1:**
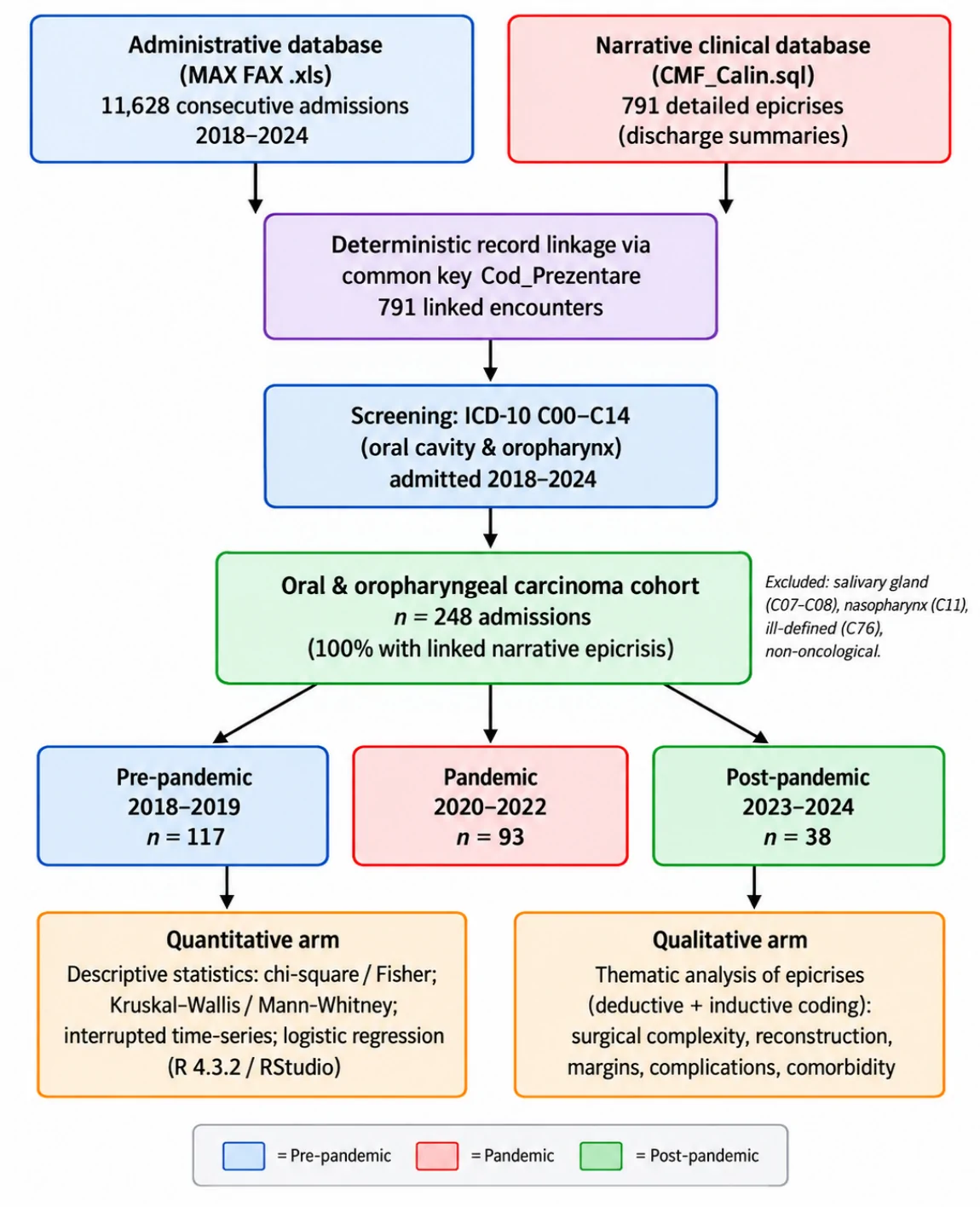
Study flow and data-linkage diagram. The administrative database (11,628 admissions) and the narrative epicrisis database (791 discharge summaries) were deterministically linked through the common key Cod_Prezentare. Screening for eligible oral-cavity and oropharyngeal carcinoma codes (C00, C01, C02–C06, C09, C10, and C14) among admissions recorded in 2018–2024 yielded 248 admissions, each with a linked narrative report, which were partitioned into three periods and analyzed through parallel quantitative and qualitative arms. Colors indicate the study periods: pre-pandemic (blue), pandemic (red), and post-pandemic (green).

Two institutional datasets were used. The administrative database (exported as MAX FAX.xls) contained 11,628 consecutive admissions (2017–2024) with structured fields including a unique admission identifier, the presentation code Cod_Prezentare, demographics, county of residence derived from the national identification number, admission/discharge dates, length of stay (LOS), the principal and secondary ICD-10 diagnoses, the total number of coded diagnoses, discharging physician and vital status. The narrative database (CMF_Calin.sql) contained 791 detailed epicrises (discharge summaries), each a free-text record with full operative descriptions, extended histopathology reports (grading, keratinization pattern, perineural/bone invasion, margin and lymph-node status), serial laboratory values, comorbidities and postoperative evolution. The two sources were linked deterministically on the common key Cod_Prezentare, yielding 791 fully linked encounters with both structured and narrative content (Figure 1). The variables included in the present analysis were obtained from the structured administrative database and the linked narrative discharge summaries. Demographic variables included age at admission and sex. Pathological variables extracted from the narrative records included histological confirmation of SCC, keratinization pattern, histological grade (G1–G3), perineural invasion, bone invasion, documented margin status, and lymph-node positivity. Comorbidity variables included documented hypertension, diabetes mellitus, and cardiac disease, while the number of coded diagnoses per admission was used as an administrative indicator of overall comorbidity burden. Surgical variables included neck dissection, glossectomy, mandibulectomy, maxillectomy, flap reconstruction, plate reconstruction, tracheostomy, and documented perioperative complications. Prior radiotherapy or chemotherapy, recurrence, and explicit documentation of COVID-19 were also recorded when available.

### 2.4. Study Population and Period Definition

Eligible encounters were identified from the deterministically linked dataset. The inclusion criteria were: (1) hospital admission between 1 January 2018 and 31 December 2024; (2) a principal ICD-10 diagnosis corresponding to oral-cavity or oropharyngeal carcinoma, defined as C00 and C02–C06 for the oral cavity and C01, C09, C10, and C14 for the oropharynx; and (3) availability of a linked narrative discharge summary containing the clinical information required for the study analyses. Exclusion criteria were: (1) salivary-gland carcinomas (C07–C08); (2) nasopharyngeal carcinoma (C11); (3) ill-defined head and neck malignancies (C76); (4) non-oncological admissions; and (5) records outside the predefined study interval, including a single 2017 boundary record. These exclusions were applied to ensure a consistent anatomical and temporal study scope. The final cohort comprised 248 eligible admissions, all of which had a linked narrative discharge summary (100% linkage). Three study periods were defined a priori: pre-pandemic (2018–2019), pandemic (2020–2022), and post-pandemic (2023–2024), with March 2020 defined as the onset of the pandemic period.

Several measures were implemented to reduce potential sources of bias. Eligibility criteria, anatomical definitions, and study-period boundaries were predefined and applied consistently across all study periods. Deterministic linkage using the common admission identifier was used to reduce record-matching errors. To limit information and classification bias arising from unstructured clinical documentation, the same rule-based extraction framework was applied across the entire dataset, extraction rules were iteratively calibrated against manually reviewed discharge summaries, and ambiguous findings were coded conservatively. In the multivariable analysis, adjustment was performed for available clinically relevant covariates, including age, sex, diabetes mellitus, hypertension, and anatomical site. Nevertheless, residual confounding and bias related to the retrospective single-center design and incomplete documentation could not be fully eliminated.

### 2.5. Variable Extraction from Narrative Reports

Because surgical complexity and histopathology are recorded only in free text, we developed a reproducible, rule-based extraction pipeline [25,26,27,28,29]. Epicrisis text was normalized (diacritic folding, lower-casing) and parsed with curated regular-expression dictionaries encoding Romanian surgical and pathological terminology, generating binary indicators for: histological confirmation of SCC; keratinization; grade (G1/G2/G3); perineural and bone invasion; documented margin clearance; nodal positivity; neck dissection; glossectomy, mandibulectomy and maxillectomy; flap and plate reconstruction; tracheostomy; perioperative complications; comorbidities (hypertension, diabetes mellitus, cardiac disease); prior radiotherapy/chemotherapy; explicit COVID-19 mention; and recurrence. An exploratory composite ‘advanced-disease/complexity proxy’ combined bone or perineural invasion, nodal positivity, or major bony resection. This composite was intended to capture features associated with greater disease and operative complexity and was not considered equivalent to formal TNM staging. Extraction rules were iteratively calibrated against a manually reviewed sample of epicrises until face validity was satisfactory; ambiguous constructs were deliberately resolved conservatively. For greater methodological transparency, the extraction workflow comprised four sequential steps: (1) normalization of the narrative text, including lower-casing and diacritic folding; (2) application of curated regular-expression dictionaries for predefined surgical, pathological, and clinical concepts; (3) generation of structured binary indicators from matched concepts; and (4) iterative calibration of the extraction rules against manually reviewed discharge summaries, with ambiguous findings coded conservatively. The complete extraction dictionaries, variable-definition logic, and supporting scripts are provided in Supplementary Materials to facilitate replication. The structured output of this pipeline fed the quantitative arm, while the same epicrises were independently read for the qualitative arm.

### 2.6. Statistical Analyses

All statistical analyses were performed in R version 4.3.2 (R Foundation for Statistical Computing, Vienna, Austria) within the RStudio integrated development environment (RStudio 2023.12; Posit, Boston, MA, USA). The principal packages were tidyverse and janitor (data management), gtsummary and tableone (descriptive tables), and the base stats, MASS and sandwich/lmtest packages (inferential modeling); figures were produced with ggplot2. Continuous variables are reported as median [interquartile range, IQR] and categorical variables as counts (percentages). Between-period comparisons used the χ^2^ test or Fisher’s exact test for categorical variables and the Kruskal–Wallis test (with Mann–Whitney U for pairwise pre-vs-pandemic contrasts) for continuous variables. A two-sided *p* < 0.05 was considered statistically significant; given the exploratory, hypothesis-generating nature of the study, *p*-values were not adjusted for multiplicity and are interpreted accordingly. Because this retrospective study included all eligible admissions during the predefined study period, no a priori sample-size calculation was performed. A post hoc sensitivity analysis was therefore conducted for the comparison of neck-dissection proportions between the pre-pandemic and combined pandemic/post-pandemic periods. With 117 and 131 observations, respectively, a two-sided α of 0.05 provided approximately 59% power to detect the observed difference in neck-dissection proportions (23.9% vs. 36.6%). At 80% power, the available sample size would have been sufficient to detect an increase from 23.9% to approximately 40.5%. These estimates are provided as a sensitivity assessment of the fixed retrospective sample and should not be interpreted as a substitute for prospective sample-size planning.

Temporal dynamics of monthly SCC admissions (84 months, January 2018–December 2024) were modeled by segmented interrupted time-series regression using a Poisson generalized linear model, following established methodological recommendations [30], with the pandemic onset (March 2020) as the interruption point. The model included a baseline time trend, an immediate level change, and a post-interruption slope change; a counterfactual (extrapolation of the pre-pandemic trend) was computed for visualization. Predictors of neck dissection were examined using multivariable logistic regression. The pandemic period was the exposure of interest, while age, sex, diabetes mellitus, hypertension, and anatomical site were included as available potential confounding factors. Other clinically relevant potential confounders, including tobacco and alcohol exposure, socioeconomic status, referral and treatment delays, access to healthcare, formal tumor stage, and treatment availability, could not be incorporated because these variables were not consistently available in the retrospective records. Results are expressed as adjusted odds ratios (ORs) and 95% confidence intervals (CIs). The complete statistical-analysis scripts are provided in Supplementary Materials.

### 2.7. Qualitative Thematic Analysis

The qualitative arm applied reflexive thematic analysis to the full set of 248 linked discharge summaries using a hybrid deductive–inductive framework [31]. The coding process was conducted by a single researcher (C.M.), the statistician responsible for data linkage, narrative-data mining, and statistical analysis in the study. Deductive codes were defined a priori based on the surgical-oncology literature and included surgical complexity, reconstruction strategy, margin clearance, complications, comorbidity burden, and advanced-disease proxies. During close reading of the discharge summaries, additional inductive codes were identified and incorporated into the framework, including concepts related to the management of intraoperative complications and multimorbidity-driven perioperative optimization. The coding framework was iteratively refined throughout the review process as recurrent concepts were identified and existing categories were clarified. Coding was organized in a structured matrix, and representative fully anonymized excerpts were translated from Romanian for presentation. Because coding was performed by a single researcher, no inter-rater reliability assessment was applicable. The rule-based indicators and thematic codes were subsequently interpreted jointly to integrate quantitative frequencies with qualitative contextual information.

## 3. Results

The results are presented in the following sequence: cohort selection and demographic characteristics, temporal changes in admission volume, surgical and histopathological complexity, comorbidity burden, interrupted time-series analysis, multivariable regression, and qualitative thematic findings.

### 3.1. Cohort Overview and Demographic Profile

The final cohort comprised 248 admissions for oral and oropharyngeal carcinoma over 2018–2024: 117 (47.2%) in the pre-pandemic period, 93 (37.5%) during the pandemic period, and 38 (15.3%) in the post-pandemic period. Among these admissions, 180/248 (72.6%) were recorded for male patients, corresponding to a male-to-female ratio of 2.6:1. The mean age at admission was 62.6 ± 11.2 years, and 118/248 admissions (47.6%) involved patients aged ≥65 years. Age and sex distributions across admissions were broadly stable across the three study periods, with no significant between-period differences (Table 1). In contrast, two administrative markers of case complexity changed significantly: the proportion of admissions with a prolonged length of stay (≥ 7 days) rose from 43.6% (pre-pandemic) to 60.2% (pandemic) and 63.2% (post-pandemic) (*p* = 0.022), and the median number of coded diagnoses per admission—a proxy for multimorbidity and clinical complexity—increased from 4 [IQR 3–6] to 5 [IQR 5–6] (Kruskal–Wallis *p* = 0.003; pre-pandemic vs. pandemic Mann–Whitney *p* = 0.001).

### 3.2. Temporal Dynamics of Surgical Volume

**Figure 2:**
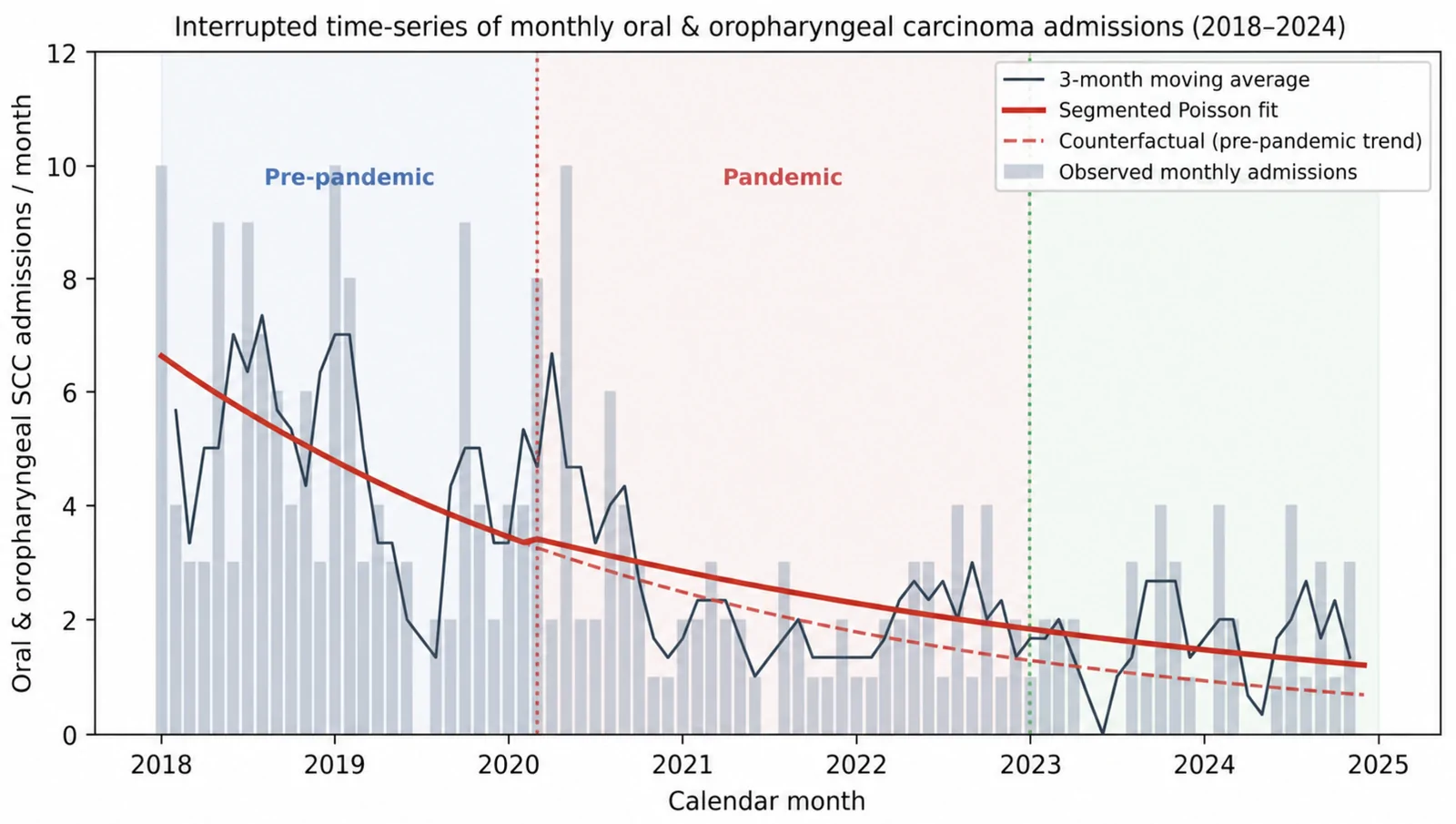
Interrupted time-series of monthly oral and oropharyngeal carcinoma admissions, January 2018–December 2024. Grey bars: observed monthly counts; dark line: 3-month moving average; solid red line: fitted segmented Poisson model; dashed red line: counterfactual extrapolation of the pre-pandemic trend. Vertical dotted lines mark the pandemic onset (March 2020) and the start of the post-pandemic period (January 2023). Monthly SCC admissions showed a significant declining baseline trend of approximately 2.7% per month, without a significant immediate level or post-onset slope change at the pandemic interruption point.

**Figure 3:**
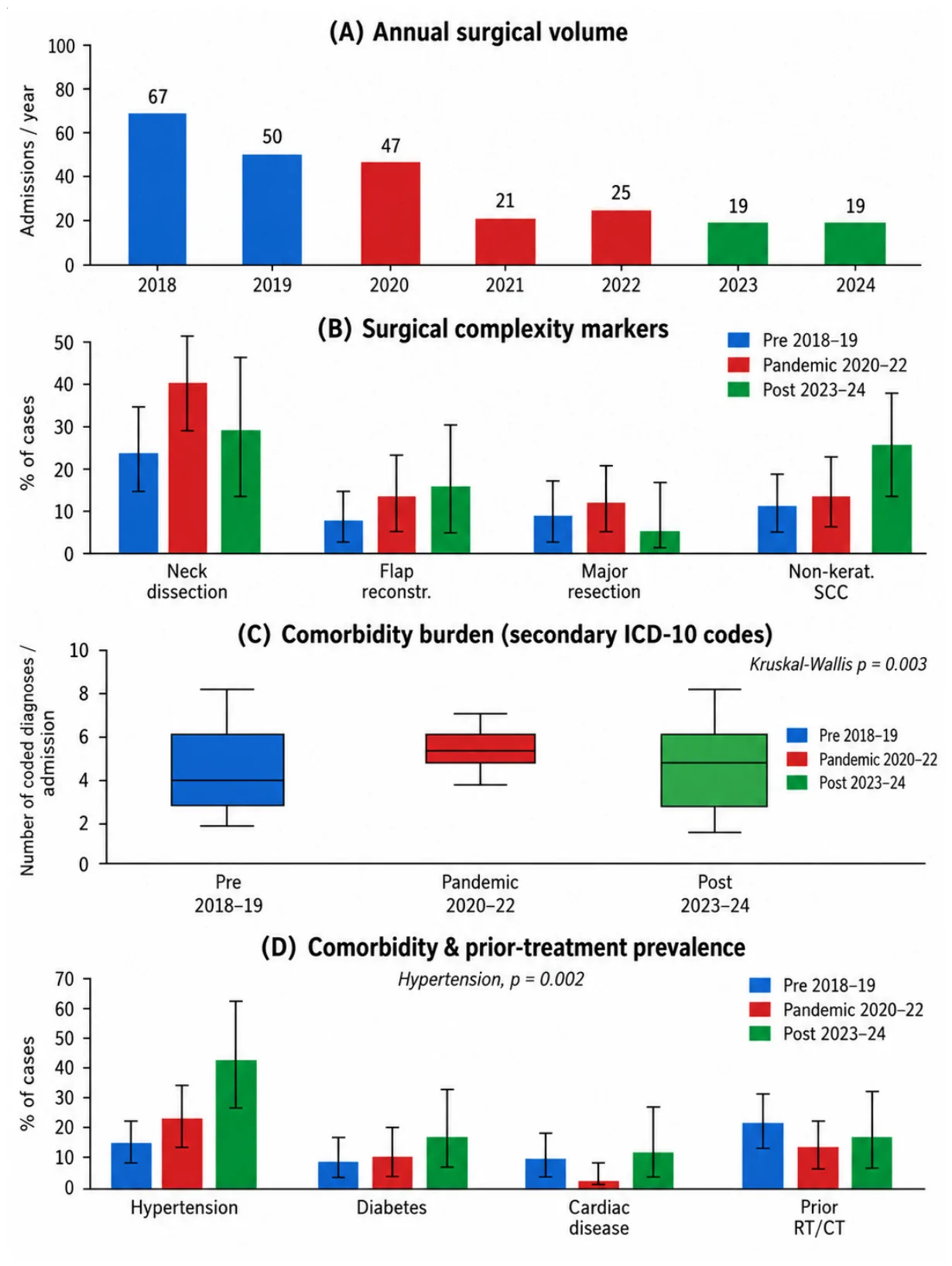
Period comparison of case-mix and burden. (**A**) Observed annual surgical admission counts, color-coded by study period; each bar represents the total number of admissions recorded in a single calendar year. (**B**) Surgical and histopathological markers across study periods; bars show proportions of admissions and error bars represent 95% confidence intervals. Neck dissection increased significantly across periods (*p* = 0.045), while flap reconstruction, major resection, and non-keratinizing SCC showed non-significant differences. (**C**) Administrative comorbidity burden, approximated by the number of coded diagnoses per admission; boxplots show the median and interquartile range (Kruskal–Wallis *p* = 0.003). (**D**) Prevalence of key comorbidities and prior oncological treatment across study periods; bars show proportions of admissions and error bars represent 95% confidence intervals. Documented hypertension increased significantly across periods (*p* = 0.002). Colors consistently indicate the study periods across all panels: blue, pre-pandemic; red, pandemic; green, post-pandemic.

Surgical volume contracted markedly and durably across the study window. Mean monthly admissions fell from 4.9 in the pre-pandemic period to 2.6 during the pandemic (a 47% relative reduction) and to 1.6 in the post-pandemic period (a 68% reduction versus baseline). Segmented Poisson interrupted time-series modeling (Figure 2, Table 2) demonstrated a significant, sustained downward baseline trend of approximately 2.7% per month (IRR 0.973, 95% CI 0.950–0.996; *p* = 0.024). Annual counts declined from 67 (2018) and 50 (2019) to 47, 21 and 25 during 2020–2022, and remained depressed at 19 per year in both 2023 and 2024 (Figure 3A). The immediate level change at the pandemic onset (March 2020) was not statistically significant (IRR 1.05, 95% CI 0.63–1.73; *p* = 0.856), and the post-interruption slope change was negligible (IRR 1.009, 95% CI 0.983–1.036; *p* = 0.497). These findings indicate a significant declining baseline trend in surgical SCC admissions over the study period, without a statistically significant immediate level change or post-pandemic slope change attributable to the onset of the pandemic. Observed admission counts remained low during 2023–2024.

### 3.3. Anatomical Subsite Distribution

The cohort was overwhelmingly oral-cavity in origin (239/248, 96.4%), with only 9 oropharyngeal cases (3.6%). The most frequent subsites were the tongue (C02, 86 cases, 34.7%), floor of mouth (C04, 52 cases, 21.0%), other or unspecified mouth (C06, 46 cases, 18.5%), lip (C00, 28 cases, 11.3%), and palate (C05, 22 cases, 8.9%). The relative subsite distribution did not differ significantly across periods (*p* = 0.65). Detailed anatomical subsite distributions are provided in Supplementary Table S1.

### 3.4. Surgical Complexity and Reconstruction

Markers of operative complexity shifted toward more extensive surgery during and after the pandemic (Figure 3B). The use of neck dissection rose significantly, from 23.9% of admissions pre-pandemic to 39.8% during the pandemic, before partially settling at 28.9% post-pandemic (χ^2^ *p* = 0.045; pre-vs-pandemic Fisher *p* = 0.016). Flap-based reconstruction increased monotonically across periods (8.5% → 14.0% → 15.8%), as did documented intraoperative margin-clearance statements (11.1% → 19.4% → 15.8%), although these trends did not reach statistical significance in this sample. Other surgical procedures and perioperative outcomes did not differ significantly across periods and are detailed in Supplementary Table S2.

### 3.5. Histopathological Features

No histopathological descriptor differed significantly across the study periods. Detailed results are provided in Supplementary Table S3.

### 3.6. Comorbidity Burden and Prior Oncological Treatment

Hypertension increased significantly across the study periods, from 14.5% before the pandemic to 21.5% during the pandemic and 42.1% in the post-pandemic period (*p* = 0.002). Explicit mention of COVID-19 in the clinical documentation was limited to the pandemic and post-pandemic periods, occurring in 30.1% and 10.5% of cases, respectively (*p* < 0.001). Cardiac disease also differed across periods (*p* = 0.036), although the pattern was non-monotonic and based on small event counts. Detailed comorbidity and prior oncological treatment data are provided in Supplementary Table S4.

### 3.7. Multivariable Predictors of Neck Dissection

In the multivariable logistic regression model, the pandemic/post-pandemic period was independently associated with higher odds of neck dissection compared with the pre-pandemic period (adjusted OR 1.87, 95% CI 1.06–3.29; *p* = 0.030), after adjustment for age, sex, diabetes mellitus, hypertension, and anatomical site. Age, sex, diabetes mellitus, hypertension, and anatomical site were not independently associated with neck dissection. The complete multivariable logistic regression results are presented in Table 2.

### 3.8. Qualitative Thematic Findings

**Figure 4:**
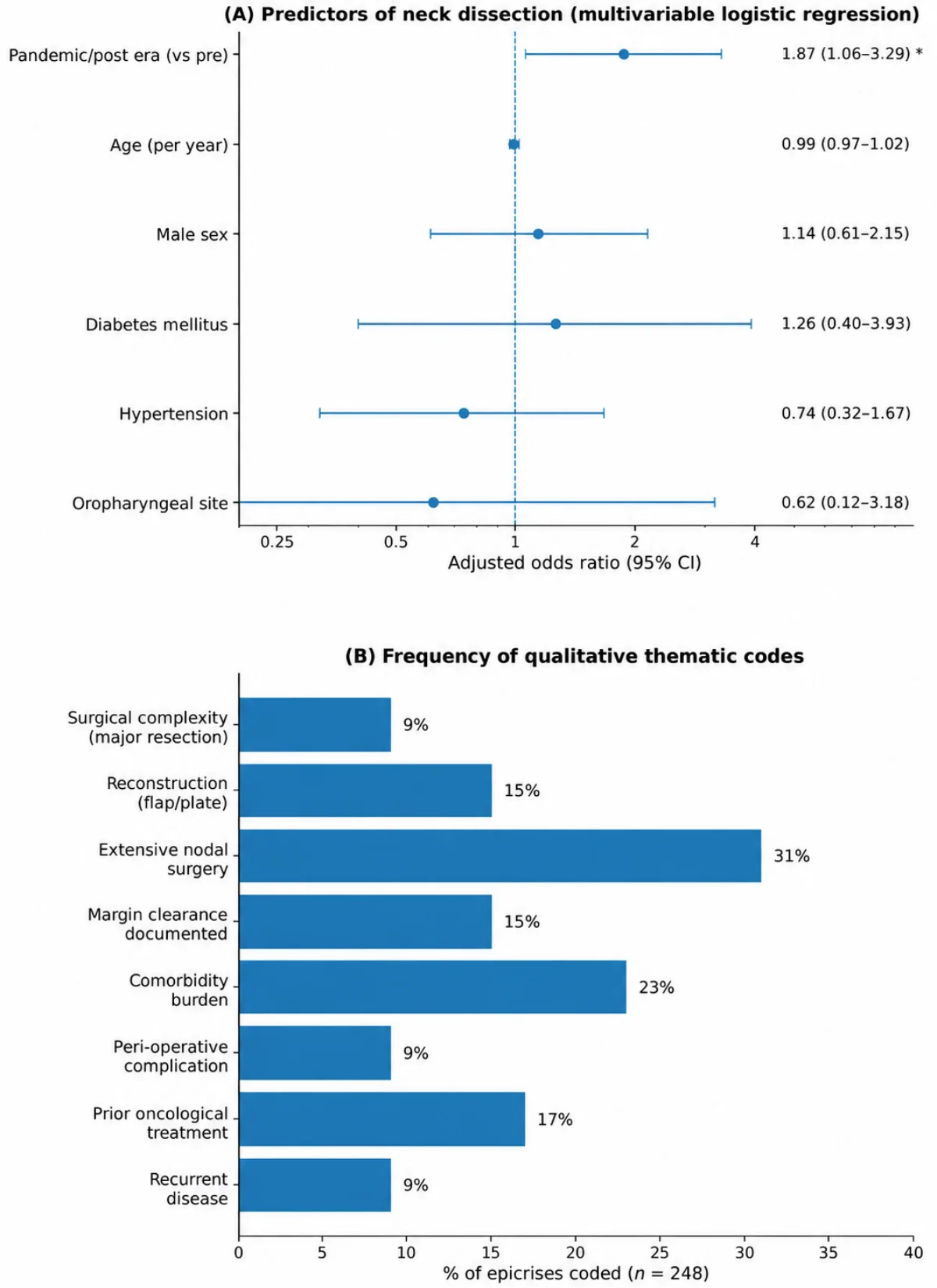
(**A**) Forest plot of adjusted odds ratios from the multivariable logistic regression model for neck dissection; the pandemic/post era was independently associated with nearly twofold higher odds (OR 1.87, 95% CI 1.06–3.29; * *p* < 0.05). (**B**) Frequency of qualitative thematic codes across the 248 linked discharge summaries, illustrating the dominance of extensive nodal surgery, comorbidity burden and reconstruction within the surgical narrative.

Thematic analysis of the 248 linked discharge summaries identified recurrent qualitative codes related to surgical complexity, reconstruction, extensive nodal surgery, margin clearance, comorbidity burden, perioperative complications, prior oncological treatment, and recurrent disease (Figure 4B). Representative anonymized excerpts and detailed thematic findings are provided in Supplementary Table S5.

## 4. Discussion

This study identified several clinically relevant changes in oral and oropharyngeal SCC admissions between 2018 and 2024. First, the interrupted time-series analysis showed a significant declining baseline monthly trend in surgical admissions, without a significant immediate level or slope change at the onset of the pandemic. Second, patients treated during the pandemic and post-pandemic periods had significantly higher odds of undergoing neck dissection after adjustment for age, sex, diabetes mellitus, hypertension, and anatomical site. Third, hypertension became more frequently documented across the study periods, while qualitative analysis identified recurrent codes related to surgical complexity, reconstruction, extensive nodal surgery, comorbidity burden, perioperative complications, prior oncological treatment, and recurrent disease. These findings are discussed sequentially below in relation to the available literature.

Our findings regarding surgical admission volume are consistent with international evidence showing substantial disruption of cancer surgery during the COVID-19 pandemic. The COVIDSurg Collaborative reported that a proportion of patients affected by lockdown restrictions did not undergo planned cancer surgery [11], while modeling studies projected excess cancer mortality associated with diagnostic and treatment delays [10]. A 2023 systematic review and meta-analysis similarly identified a significant reduction in the number of head and neck cancer procedures performed during the pandemic compared with the pre-pandemic period [13]. In our cohort, the interrupted time-series analysis demonstrated a significant declining baseline monthly trend, but no significant immediate level change or post-onset slope change attributable specifically to the pandemic. This pattern suggests that the reduction in surgical admissions may reflect a longer-term structural trend, potentially amplified by pandemic-related disruption, rather than an isolated effect occurring exclusively at the onset of the pandemic. The persistence of low admission numbers during 2023–2024 further suggests that surgical volume had not returned to pre-pandemic levels. Importantly, our time-series findings differ in one respect from reports describing an abrupt pandemic-associated reduction in oncological activity. Although the crude admission counts in our cohort decreased markedly during the pandemic, segmented analysis did not identify a statistically significant immediate level change or a change in slope at the March 2020 interruption point. This distinction is clinically and methodologically relevant, because it suggests that the lower surgical volume observed during the pandemic occurred against an already declining institutional trend rather than representing a discrete pandemic-onset effect. Nevertheless, the magnitude and persistence of the reduction are broadly consistent with the international literature documenting disruption of head and neck cancer diagnosis and treatment during the pandemic [11,13,14,15,16,17,18,19]. The continued low volume in 2023–2024 also extends previous observations, many of which were restricted to the early pandemic period, and indicates that recovery of surgical activity at our center was incomplete within the study window.

The second major finding was the increase in surgical complexity, particularly the higher likelihood of neck dissection among patients treated during and after the pandemic. Neck dissection increased from 23.9% before the pandemic to 39.8% during the pandemic, and the pandemic/post-pandemic period remained independently associated with neck dissection after adjustment for potential confounders. This finding indicates greater surgical extent among patients who reached surgery; however, because of the observational design, incomplete standardized TNM staging, and unavailable HPV and risk-factor data, it should not be interpreted as evidence that the pandemic caused increased tumor severity or surgical complexity. Previously reported associations between diagnostic or treatment delays and more advanced presentation provide a plausible explanation for this pattern [6,7,8,9].

Our findings are also consistent with single-center head and neck oncology series reported during the pandemic [12,14,16,17,18,19]. Tevetoğlu et al. described a higher frequency of T3–T4 tumors, more advanced nodal disease in oral-cavity cancer, and an increased need for flap reconstruction [12]. Similarly, Zubair et al. reported an increase in advanced-stage presentation, together with longer referral-to-treatment intervals and a greater requirement for airway-stabilization procedures [14]. Other studies have also reported changes in disease extent, staging, and reconstructive requirements during the pandemic [16,17,18,19]. In the present study, the increased odds of neck dissection, together with the observed trend toward greater use of flap reconstruction, provide a coherent signal of increased operative complexity among patients receiving surgical treatment. Nevertheless, these findings should not be interpreted as direct evidence of stage migration because formal TNM staging was not uniformly available.

These comparisons should, however, be interpreted with caution. Tevetoğlu et al. directly documented a greater frequency of T3–T4 tumors and more advanced nodal disease, whereas Zubair et al. reported a higher proportion of advanced-stage presentations and longer referral-to-treatment intervals [12,14]. In contrast, the present study did not have sufficiently complete TNM staging or referral-delay data to reproduce these endpoints directly. Our principal signal was instead an increase in neck dissection from 23.9% before the pandemic to 39.8% during the pandemic, together with higher adjusted odds of neck dissection in the combined pandemic/post-pandemic period. Thus, our findings are directionally consistent with reports of increased treatment complexity during the pandemic, but they provide evidence of greater operative extent rather than direct evidence of more advanced tumor stage. The non-significant increase in flap reconstruction observed in our cohort further supports this cautious interpretation and is broadly compatible with the greater reconstructive requirements reported in other head and neck oncology series [12,14,16,17,18,19].

Beyond the changes in surgical volume and operative complexity, the study identified a significant increase in documented hypertension across the three periods, from 14.5% before the pandemic to 21.5% during the pandemic and 42.1% in the post-pandemic period. This pattern may reflect a greater cardiovascular comorbidity burden among patients treated in the later periods, changes in the case mix of patients reaching surgical treatment, or more complete documentation of pre-existing conditions over time. Frailty is a well-recognized determinant of perioperative risk in patients undergoing major head and neck oncological surgery, and previous studies have demonstrated associations between increasing frailty burden and adverse postoperative outcomes [32,33,34,35,36,37]. Although the present study cannot determine whether the observed increase in hypertension reflects a true temporal increase in prevalence or differences in referral and documentation patterns, the finding reinforces the importance of systematic cardiovascular assessment, preoperative risk stratification, and coordinated perioperative management in medically complex patients.

The qualitative findings complement the quantitative results by showing that the clinical narratives frequently contained codes related to extensive nodal surgery, reconstruction, margin clearance, comorbidity burden, and perioperative complications. These patterns suggest that surgical complexity extended beyond the binary outcome of neck dissection and involved broader reconstructive and perioperative demands. This interpretation is consistent with previous reports of more advanced presentation and increased reconstructive needs during the pandemic [12,14,16,17,18,19]. However, because the thematic codes were derived from non-standardized discharge summaries and were not equivalent to formal TNM staging or prospectively collected measures of surgical complexity, they should be interpreted as contextual evidence rather than independent proof of stage migration.

The findings of the present study have several practical implications for clinical care and health-service planning. First, the persistent reduction in surgical admissions supports active case finding rather than passive waiting. Structured recall and triage protocols for patients with high-risk oral lesions, together with primary-care education regarding early referral, may help identify patients whose diagnosis or treatment has been delayed [7,8,9,38,39,40,41]. Second, the observed increase in surgical complexity and comorbidity burden may have important resource implications. Our findings suggest that reconstructive capacity, multidisciplinary prehabilitation, and perioperative medical optimization may remain important during health-system recovery and future periods of disruption, although this observation requires confirmation in larger multicenter studies [32,33,34,35,36,37,42]. Third, the observed increase in documented comorbidity burden suggests a potential role for integrated multidisciplinary pathways in the management of older and medically complex patients undergoing oral and maxillofacial oncological surgery; however, broader implications should be confirmed in multicenter studies. In this context, diabetes mellitus also warrants particular attention, given its systemic and oral-health implications; recent evidence from Romanian patients with type 2 diabetes has demonstrated associations between lifestyle factors, oral health behaviors, and glycemic control, further supporting an integrated approach to the management of medically complex patients [43].

From an informatics perspective, the study also illustrates the potential value of routinely collected clinical data for longitudinal oncological surveillance. Deterministic linkage between structured administrative data and narrative discharge summaries enabled quantitative epidemiological indicators to be interpreted alongside detailed operative, pathological, and perioperative information. The rule-based extraction approach may be transferable to other centers and to future datasets. At the same time, the unstructured and non-standardized nature of the discharge summaries limited consistent extraction of some histopathological and operative variables. The adoption of standardized, machine-readable operative and histopathological reporting templates could therefore improve prospective surveillance, facilitate multicenter comparisons, and strengthen future quality registries.

This study has several strengths. It covers a seven-year period encompassing the pre-pandemic, pandemic, and post-pandemic phases and combines structured administrative data with detailed narrative clinical information. The mixed-methods design allowed quantitative findings to be contextualized through qualitative thematic analysis, while the rule-based extraction pipeline and transparent statistical approach support reproducibility and potential transfer to other centers. The integration of routinely collected structured and narrative data also illustrates the potential of hospital information systems for longitudinal oncological surveillance.

Several limitations should be considered. The retrospective, single-center design introduces several potential sources of bias, including selection bias related to referral patterns, information and classification bias arising from non-standardized clinical documentation, and residual confounding due to unavailable clinical and healthcare-access variables. First, this was a single-center retrospective study conducted at a tertiary referral institution. Changes in referral patterns during the pandemic may have selectively concentrated more complex oncological cases at our center, introducing referral bias and potentially influencing the observed differences in surgical complexity. Consequently, the findings may not be fully generalizable to other institutions or to population-level trends and require confirmation in multicenter studies. Furthermore, the retrospective nature of the study entails inherent risks of incomplete or inconsistently documented clinical data and residual confounding, as the available records did not permit comprehensive adjustment for all potentially relevant factors. Several potentially relevant confounding factors, including tobacco and alcohol exposure, socioeconomic status, referral and treatment delays, access to healthcare, formal tumor stage, and treatment availability, were not consistently available in the retrospective records and therefore could not be incorporated into the multivariable model. Consequently, the observed association between the pandemic/post-pandemic period and neck dissection should be interpreted as an adjusted association rather than as evidence of a causal effect of the pandemic. Moreover, the interrupted time-series analysis identified a significant pre-existing downward trend in surgical admissions, without a statistically significant immediate level change or post-interruption slope change. Therefore, the observed decline in admissions cannot be confidently attributed to the COVID-19 pandemic itself and should be interpreted as a temporal association. Second, tumor stage was inferred from narrative histopathological and surgical proxies rather than from uniformly documented pathological TNM staging. These proxy measures are not equivalent to formal TNM classification and may be subject to classification bias. Therefore, the observed increase in neck dissection should be interpreted as a marker of greater operative complexity rather than as definitive evidence of pandemic-related stage migration. Third, the post-pandemic subgroup was relatively small (*n* = 38), reducing the precision of estimates for 2023–2024 and limiting statistical power for analyses specific to this period. Accordingly, findings involving the post-pandemic subgroup should be interpreted cautiously. The fixed retrospective sample also provided limited statistical power for detecting moderate between-period differences, particularly in subgroup analyses; therefore, non-significant findings should not necessarily be interpreted as evidence of equivalence. Fourth, the oropharyngeal subgroup was also very small (9/248, 3.6%), which precluded meaningful separate analysis and limited direct comparisons between oral and oropharyngeal SCC. Fifth, human papillomavirus (HPV) status was not systematically documented. Given the important epidemiological and prognostic role of HPV in oropharyngeal SCC, this limitation precluded HPV-based stratification and further restricted interpretation of the oropharyngeal subgroup. Sixth, multiple statistical comparisons were performed without formal adjustment for multiplicity, which increases the risk of false-positive findings. Accordingly, statistically significant findings, particularly those close to the conventional significance threshold, should be interpreted cautiously and considered exploratory. The qualitative coding was performed by a single researcher, which may have introduced interpretive bias and precluded assessment of inter-rater reliability. Finally, the discharge summaries were unstructured and non-standardized, limiting the consistent capture of rare clinical, histopathological, and operative descriptors. Inconsistent documentation may therefore have resulted in missing or misclassified information despite conservative extraction rules. The adoption of standardized, machine-readable operative and pathology templates could improve data completeness, reduce the risk of misclassification, and strengthen future prospective surveillance and quality registries.

Future investigations should address these limitations through multicenter, prospectively designed studies using standardized data-collection protocols. In particular, systematic documentation of TNM stage, HPV status, tobacco and alcohol exposure, socioeconomic factors, referral and treatment intervals, and healthcare-access variables would allow more complete adjustment for confounding and more direct assessment of stage migration. Such standardized reporting would also reduce information and classification bias associated with unstructured clinical documentation. Larger multicenter cohorts would also improve statistical power, particularly for post-pandemic and anatomical subgroup analyses, while independent coding by multiple researchers could reduce interpretive bias in qualitative analyses and permit assessment of inter-rater reliability. Finally, longer follow-up beyond 2024 would help determine whether the observed changes in surgical volume and case complexity represent persistent trends or delayed recovery of oncological services.

## 5. Conclusions

This study identified a significant declining baseline trend in oral and oropharyngeal SCC surgical admissions between 2018 and 2024, without a significant immediate level or slope change specifically attributable to the onset of the COVID-19 pandemic. Patients treated during the pandemic and post-pandemic periods had higher adjusted odds of undergoing neck dissection. Qualitative findings provided additional context regarding surgical complexity, reconstruction, comorbidity burden, and perioperative management. These findings should be interpreted as temporal and adjusted associations rather than evidence of a direct causal effect of the COVID-19 pandemic.

These findings may inform the design of future multicenter prospective studies incorporating standardized TNM staging, HPV status, relevant behavioral and healthcare-access factors, and longer post-pandemic follow-up to determine whether the observed changes in surgical volume and operative complexity persist over time and are reproducible across different healthcare settings.

## Figures and Tables

**Table 1:** Demographic and administrative characteristics of oral and oropharyngeal carcinoma admissions by pandemic period.

Characteristic	Pre-pandemic (2018–2019) *n* = 117	Pandemic (2020–2022) *n* = 93	Post-pandemic (2023–2024) *n* = 38	*p*-Value
**Age at admission, median [IQR], years**	65 [56–71]	62 [55–68]	60 [55–71]	0.310
**Admissions involving patients aged ≥ 65 years, *n* (%)**	61 (52.1)	41 (44.1)	16 (42.1)	0.389
**Admissions involving male patients, *n* (%)**	82 (70.1)	73 (78.5)	25 (65.8)	0.237
**Length of stay, median [IQR], days**	4.2 [2.0–13.9]	8.0 [4.2–11.1]	7.6 [5.4–10.8]	0.245
**Length of stay ≥ 7 days, *n* (%)**	51 (43.6)	56 (60.2)	24 (63.2)	0.022
**Coded diagnoses, median [IQR], *n***	4 [3–6]	5 [5–6]	5 [3–6]	0.003
**Mean monthly admissions**	4.9	2.6	1.6	—

IQR, interquartile range. The unit of analysis was the hospital admission. *p*-values: χ^2^ test (categorical), Kruskal–Wallis test (continuous). Mean monthly admissions were computed over 24, 36, and 24 months, respectively.

**Table 2:** Interrupted time-series and multivariable logistic regression results.

Model/Parameter	Estimate	95% CI	*p*-Value
**Interrupted time-series (segmented Poisson GLM)**			
** Baseline monthly trend (IRR)**	0.973	0.950–0.996	0.024
** Level change at pandemic onset (IRR)**	1.048	0.633–1.733	0.856
** Slope change after onset (IRR)**	1.009	0.983–1.036	0.497
**Multivariable logistic regression for neck dissection (adjusted OR)**			
** Pandemic/post-pandemic period (vs. pre-pandemic period)**	1.87	1.06–3.29	0.030
** Age (per year)**	0.99	0.97–1.02	0.550
** Male sex**	1.14	0.61–2.15	0.682
** Diabetes mellitus**	1.26	0.40–3.93	0.694
** Hypertension**	0.74	0.32–1.67	0.462
** Oropharyngeal site (vs oral cavity)**	0.62	0.12–3.18	0.568

IRR, incidence rate ratio; OR, odds ratio; CI, confidence interval; GLM, generalized linear model. The logistic regression model included 76 neck-dissection events among 248 admissions.

## Data Availability

The individual-level clinical data are not publicly available because they contain potentially identifying information. De-identified derived data and the scripts used for cohort definition, record linkage, narrative extraction, statistical analysis, validation, and figure generation are provided in Supplementary Materials. Additional information may be obtained from the corresponding author upon reasonable request, subject to institutional data-governance approval.

## Supplementary Materials

The following supporting information can be downloaded at https://www.mdpi.com/article/10.3390/jcm15186963/s1. Supplementary Table S1: Anatomical subsite distribution by pandemic period, *n* (% of period); Supplementary Table S2: Surgical procedures and complexity markers extracted from narrative operative reports, by period, *n* (%); Supplementary Table S3: Histopathological features extracted from narrative reports, by period, *n* (%); Supplementary Table S4: Comorbidity profile and prior oncological treatment by study period, *n* (%); Supplementary Table S5: Representative anonymized excerpts and qualitative themes identified in the linked discharge summaries. Supplementary Materials contains the supplementary tables, derived analytical datasets, analytical and data-processing scripts, and figure-generation code.
