# Supplementary material for: Epidemiological Assessment of Oral and Oropharyngeal Squamous Cell Carcinomas Before, During, and After the COVID-19 Pandemic, 2018–2024
Source: J Clin Med. 2026 Sep 8;15(18):6963. doi: 10.3390/jcm15186963 (PMC13607342; doi:10.3390/jcm15186963)
Supplement: Supplementary file 1 [file jcm-15-06963-s001.zip › tables/Supplementary_Table_S1.pdf]

Supplementary Table S1. Anatomical subsite distribution by pandemic period, n (% of period)

**Subsite (ICD-10)**	**Pre n = 117**	**Pandemic n = 93**	**Post n = 38**	**Total n = 248**
Tongue (C02)	52 (44.4)	28 (30.1)	6 (15.8)	86 (34.7)
Floor of mouth (C04)	23 (19.7)	19 (20.4)	10 (26.3)	52 (21.0)
Other or unspecified mouth (C06)	19 (16.2)	18 (19.4)	9 (23.7)	46 (18.5)
Lip (C00)	9 (7.7)	12 (12.9)	7 (18.4)	28 (11.3)
Palate (C05)	7 (6.0)	12 (12.9)	3 (7.9)	22 (8.9)
Gum (C03)	3 (2.6)	0 (0.0)	2 (5.3)	5 (2.0)
Oropharynx (C01/C09/C10/C14)	4 (3.4)	4 (4.3)	1 (2.6)	9 (3.6)

Values are n (column %). Overall distribution across periods: χ^2 p = 0.65; oropharyngeal vs oral-cavity proportion p = 0.89.
