# Supplementary material for: Epidemiological Assessment of Oral and Oropharyngeal Squamous Cell Carcinomas Before, During, and After the COVID-19 Pandemic, 2018–2024
Source: J Clin Med. 2026 Sep 8;15(18):6963. doi: 10.3390/jcm15186963 (PMC13607342; doi:10.3390/jcm15186963)
Supplement: Supplementary file 1 [file jcm-15-06963-s001.zip › tables/Supplementary_Table_S2.pdf]

Supplementary Table S2. Surgical procedures and complexity markers extracted from narrative operative reports, by period, n (%).

**Procedure / complexity marker**	**Pre**<br>**n = 117**	**Pandemic**<br>**n = 93**	**Post**<br>**n = 38**	**p-value**
Neck dissection	28 (23.9)	37 (39.8)	11 (28.9)	0.045
Glossectomy / partial tongue resection	26 (22.2)	16 (17.2)	7 (18.4)	0.646
Mandibular resection	5 (4.3)	8 (8.6)	1 (2.6)	0.274
Maxillary resection	5 (4.3)	2 (2.2)	1 (2.6)	0.671
Major bony resection (composite)	10 (8.5)	10 (10.8)	2 (5.3)	0.596
Flap reconstruction	10 (8.5)	13 (14.0)	6 (15.8)	0.331
Plate / osteosynthesis reconstruction	4 (3.4)	4 (4.3)	0 (0.0)	0.444
Tracheostomy	2 (1.7)	2 (2.2)	0 (0.0)	0.671
Perioperative complication	11 (9.4)	7 (7.5)	4 (10.5)	0.828

p-values from χ^2 test across the three periods. The composite major bony resection denotes mandibulectomy or maxillectomy.
