# Supplementary material for: Epidemiological Assessment of Oral and Oropharyngeal Squamous Cell Carcinomas Before, During, and After the COVID-19 Pandemic, 2018–2024
Source: J Clin Med. 2026 Sep 8;15(18):6963. doi: 10.3390/jcm15186963 (PMC13607342; doi:10.3390/jcm15186963)
Supplement: Supplementary file 1 [file jcm-15-06963-s001.zip › tables/Supplementary_Table_S3.pdf]

Supplementary Table S3. Histopathological features extracted from narrative reports, by period, n (%).

**Histopathological feature**	**Pre n = 117**	**Pandemic n = 93**	**Post n = 38**	**p-value**
SCC histologically confirmed	73 (62.4)	54 (58.1)	29 (76.3)	0.144
Non-keratinizing pattern	14 (12.0)	13 (14.0)	10 (26.3)	0.093
Moderate–poor grade (G2–G3)	16 (13.7)	20 (21.5)	10 (26.3)	0.143
Perineural invasion	1 (0.9)	3 (3.2)	0 (0.0)	0.276
Bone invasion	3 (2.6)	0 (0.0)	0 (0.0)	0.183
Nodal positivity documented	10 (8.5)	6 (6.5)	0 (0.0)	0.176
Margin clearance documented	13 (11.1)	18 (19.4)	6 (15.8)	0.246

Indicators are derived from free-text histopathology sections and are conservatively coded; absolute frequencies are therefore lower-bound estimates of true prevalence (see Limitations).
