# Supplementary material for: Epidemiological Assessment of Oral and Oropharyngeal Squamous Cell Carcinomas Before, During, and After the COVID-19 Pandemic, 2018–2024
Source: J Clin Med. 2026 Sep 8;15(18):6963. doi: 10.3390/jcm15186963 (PMC13607342; doi:10.3390/jcm15186963)
Supplement: Supplementary file 1 [file jcm-15-06963-s001.zip › tables/Supplementary_Table_S4.pdf]

Supplementary Table S4. Comorbidity profile and prior oncological treatment by study period, n (%).

**Comorbidity / treatment history**	**Pre n = 117**	**Pandemic n = 93**	**Post n = 38**	**p-value**
Hypertension	17 (14.5)	20 (21.5)	16 (42.1)	0.002
Diabetes mellitus	9 (7.7)	8 (8.6)	6 (15.8)	0.314
Cardiac disease	10 (8.5)	1 (1.1)	4 (10.5)	0.036
Prior radiotherapy / chemotherapy	25 (21.4)	12 (12.9)	6 (15.8)	0.264
Documented recurrence	9 (7.7)	13 (14.0)	1 (2.6)	0.091
Explicit COVID-19 mention	0 (0.0)	28 (30.1)	4 (10.5)	<0.001

p-values from χ^2 test. The non-monotonic cardiac-disease pattern reflects small event counts and is interpreted cautiously.
