# Supplementary material for: Epidemiological Assessment of Oral and Oropharyngeal Squamous Cell Carcinomas Before, During, and After the COVID-19 Pandemic, 2018–2024
Source: J Clin Med. 2026 Sep 8;15(18):6963. doi: 10.3390/jcm15186963 (PMC13607342; doi:10.3390/jcm15186963)
Supplement: Supplementary file 1 [file jcm-15-06963-s001.zip › tables/Supplementary_Table_S5.pdf]

Supplementary Table S5. Representative anonymized excerpts and qualitative themes identified in the linked discharge summaries.

**Theme**	**Representative anonymized excerpt (translated)**	**n (%)**
Surgical complexity / multi-compartment resection	"…radical tumor excision with oncological safety margins of the right tongue margin and base, ipsilateral tonsil en bloc with a mandibular segment…"	—
Reconstruction strategy	"…the mandibular arch continuity defect is reconstructed with a reconstruction plate…"; "…coverage of the orbital roof by a left temporal myofascial flap…"	≈18%
Advanced local invasion	"…non-keratinizing G2 squamous cell carcinoma of the left floor of mouth with mandibular bone invasion and suspected submandibular gland involvement…"	—
Margin-oriented oncological intent	"…excision with oncological safety margins…"; "…excised in healthy tissue…"	≈15%
Multimorbidity-driven optimization	"…patient with multiple comorbidities (ischemic cardiomyopathy, hypertension, insulin-requiring diabetes)… preoperative medical optimization instituted…"	≈30%
Complication management	"…postoperative oral wound dehiscence at the pelvi-linguo-mandibular sulcus…"; "…pharyngeal-wound dehiscence with salivary drainage, resolved by discharge…"	≈9%

Excerpts were translated from Romanian and de-identified. Frequencies (where shown) correspond to the rule-based indicators in Figure 4B; em dashes denote themes captured qualitatively but not reduced to a single indicator.
